# Cyanogenic Glucosides and Derivatives in Almond and Sweet Cherry Flower Buds from Dormancy to Flowering

**DOI:** 10.3389/fpls.2017.00800

**Published:** 2017-05-19

**Authors:** Jorge Del Cueto, Irina A. Ionescu, Martina Pičmanová, Oliver Gericke, Mohammed S. Motawia, Carl E. Olsen, José A. Campoy, Federico Dicenta, Birger L. Møller, Raquel Sánchez-Pérez

**Affiliations:** ^1^Department of Plant Breeding, CEBAS-CSICMurcia, Spain; ^2^Plant Biochemistry Laboratory, Department of Plant and Environmental Sciences, University of CopenhagenFrederiksberg, Denmark; ^3^VILLUM Research Center for Plant Plasticity, University of CopenhagenFrederiksberg, Denmark; ^4^UMR 1332 BFP, INRA, University of BordeauxVillenave d’Ornon, France

**Keywords:** amygdalin, dormancy, flowering time, LC-MS/MS, prunasin, prunasin derivatives, qRT-PCR

## Abstract

Almond and sweet cherry are two economically important species of the *Prunus* genus. They both produce the cyanogenic glucosides prunasin and amygdalin. As part of a two-component defense system, prunasin and amygdalin release toxic hydrogen cyanide upon cell disruption. In this study, we investigated the potential role within prunasin and amygdalin and some of its derivatives in endodormancy release of these two *Prunus* species. The content of prunasin and of endogenous prunasin turnover products in the course of flower development was examined in five almond cultivars – differing from very early to extra-late in flowering time – and in one sweet early cherry cultivar. In all cultivars, prunasin began to accumulate in the flower buds shortly after dormancy release and the levels dropped again just before flowering time. In almond and sweet cherry, the turnover of prunasin coincided with increased levels of prunasin amide whereas prunasin anitrile pentoside and β-D-glucose-1-benzoate were abundant in almond and cherry flower buds at certain developmental stages. These findings indicate a role for the turnover of cyanogenic glucosides in controlling flower development in *Prunus* species.

## Introduction

Cyanogenic glucosides (CNglcs) are defense compounds present in more than 3,000 plant species (Gleadow and Møller, 2014) including economically important fruit trees such as almond (*Prunus dulcis* Miller D.A. Webb syn. *P. amygdalus* Batsch) and sweet cherry (*P. avium* L.). Both fruit trees contain the phenylalanine-derived CNglcs prunasin and amygdalin. Prunasin is a β-D-monoglucoside of *R*-mandelonitrile (Kuroki and Poulton, 1987; Swain et al., 1992; Hu and Poulton, 1999; Neilson et al., 2011) and a precursor for the diglucoside amygdalin in which the two glucose moieties are β-(1→6) linked (gentiobiose). In the bitter-kernelled almond cultivars, prunasin is present in the tegument, endosperm, nucella, and cotyledons at the early stages of seed development (Frehner et al., 1990; Dicenta et al., 2002; Sánchez-Pérez et al., 2008). Amygdalin accumulates at the later state of fruit kernel development (Sánchez-Pérez et al., 2008) where its content in the kernel is around 100-fold higher compared to prunasin (Dicenta et al., 2002; Sánchez-Pérez et al., 2008). Conversely, prunasin is present in high amounts compared to amygdalin in the vegetative parts of the almond tree such as leaf, petiole, stem, and root – with no major differences in the ratios observed between sweet and bitter cultivars. Both CNglcs are synthesized *de novo* in the kernel but only amygdalin is accumulated in bitter kernels (Sánchez-Pérez et al., 2008). In sweet cherry, prunasin is present in flowers, fruits, stems, and seeds, whilst amygdalin is present in fruits and seeds only (Nahrstedt, 1972).

**FIGURE 1:**
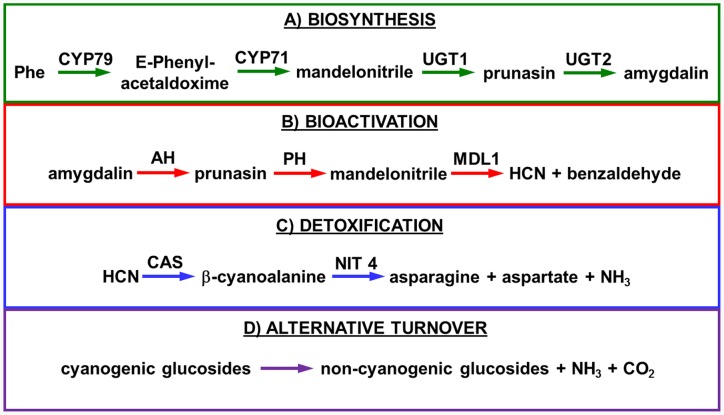
**Biosynthesis** **(A)**, bioactivation **(B)**, detoxification **(C)**, and alternative turnover **(D)** of prunasin and amygdalin in almond and sweet cherry. CYP79 and CYP71: cytochromes P450; UGT1 and UGT2: UDP-glucosyltransferases 1 and 2, respectively; AH, amygdalin hydrolase; PH, prunasin hydrolase; Phe, phenylalanine; MDL1, mandelonitrile lyase 1; β-CAS, β-cyanoalanine synthase; NIT4, nitrilase 4.

Biosynthesis of prunasin and amygdalin (**Figure 1**) involves the initial conversion of L-phenylalanine (Phe) (Mentzer and Favrebonvin, 1961) into mandelonitrile by the action of the two cytochromes P450 called CYP79D16 and CYP71AN24, recently characterized in Japanese apricot (*P. mume* Sieb. et Zucc) (Yamaguchi et al., 2014). An UDP-glucosyltransferase (UGT1, UGT85A19) catalyzes the conversion of mandelonitrile into prunasin (Franks et al., 2008). Finally, an unknown glucosyltransferase (UGT2) catalyzes the conversion of prunasin into amygdalin (**Figure 1A**).

The classic physiological function assigned to CNglcs is in chemical defense against pathogens and herbivores. This two-component defense system involves β-glucosidase and α-hydroxynitrilelyase-catalyzed hydrolysis of CNglcs resulting in the release of toxic hydrogen cyanide. The system is detonated when the CNglcs and their hydrolytic enzymes get into contact as a result of tissue and cell destruction, e.g., by herbivore attack. In this bioactivation process, amygdalin is converted into prunasin and glucose by amygdalin hydrolase (AH). Prunasin hydrolase (PH) converts prunasin into mandelonitrile and glucose (Kuroki and Poulton, 1987; Li et al., 1992; Zheng and Poulton, 1995; Zhou et al., 2002; Sánchez-Pérez et al., 2008, 2010, 2012) Mandelonitrile lyase 1 (MDL1) catalyzes the dissociation of mandelonitrile into benzaldehyde and hydrogen cyanide (Swain and Poulton, 1994a; Zheng and Poulton, 1995; Suelves and Puigdomènech, 1998; Hu and Poulton, 1999), two compounds that are bitter and toxic, respectively (Evreinoff, 1952) (**Figure 1B**).

To avoid hydrogen cyanide intoxication, plants have developed a detoxification pathway in which β-cyanoalanine synthase (β-CAS) catalyzes the conversion of hydrogen cyanide into β-cyanoalanine (**Figure 1C**). In a subsequent reaction, a type 4 nitrilase catalyzes hydration of β-cyanoalanine resulting in the production of asparagine or aspartate and ammonia (Piotrowski, 2008). Evidence for the operation of two endogenous turnover pathways for cyanogenic glucosides has recently been provided (Pičmanová et al., 2015; Nielsen et al., 2016). In both these pathways, the nitrogen of the cyanogenic glucoside is recovered as ammonia without any release of hydrogen cyanide (**Figure 1D**).

Other potential physiological functions of CNglcs include a role as transporters of carbon and nitrogen (Selmar et al., 1988), suppliers of reduced nitrogen in form of ammonia (Sánchez-Pérez et al., 2008; Nielsen et al., 2016), as modulators of oxidative stress (Møller, 2010; Neilson et al., 2013) and as regulators of seed germination (Swain and Poulton, 1994b; Pičmanová et al., 2015). Seed germination is a developmental process closely related to bud dormancy release (Wareing and Saunders, 1971; Rohde and Bhalerao, 2007). CNglcs metabolism has also been hypothesized to contribute to the nitrogen pool, thereby enabling bud opening (Gleadow and Woodrow, 2000). The levels of CNglcs and their metabolites in flower buds during endodormancy release have not previously been reported. In temperate climates, bud dormancy is the adaptive mechanism of perennial plant species to counteract the harsh environmental conditions of winter and is controlled by the required accumulation of chill and the subsequent accumulation of heat. This process enables the plant to time flowering and leafing to profit from weather conditions that are favorable for growth and development. Flowering will only happen when dormancy is broken (Fennell, 1999).

The flowering time is mainly determined by the cultivar-dependent chill requirements, with heat requirements being less important (Egea et al., 2003). The chill requirements necessary for dormancy release and flowering have been studied in *Prunus* species such as apricot (*P. armeniaca* L.) (Ruiz et al., 2007), sweet cherry (Alburquerque et al., 2008), peach (*P. persica* L.) (Weinberger, 1950), plum (*P. domestica* L.) (Okie and Hancock, 2008) and almond (Egea et al., 2003; Sánchez-Pérez et al., 2010, 2014).

When the chill requirements are low, e.g., in early-flowering cultivars, late winter or cold temperatures in spring may cause yield loss by frost (Scorza and Okie, 1991). Flowering time is one of the most important agronomic traits in almond, since late flowering cultivars counteract crop loss caused by late spring frosts (Dicenta et al., 2005). In sweet cherry, the situation is opposite, as this species has a higher range of chill requirements. Due to global warming, chill requirements are hardly fulfilled in warmer production areas (Campoy et al., 2011). Therefore, different nitrogen- or sulfur-based dormancy-breaking chemicals are applied by spraying to compensate for missing chill and to induce flowering. The most successful chemical, commercially known as Dormex^®^ (AlzChem, Trostberg, Germany), is hydrogen cyanamide (Godini et al., 2008). Hydrogen cyanamide advances flowering time up to 3 weeks and synchronizes bud break. This facilitates and advances fruit harvest as well. Even though hydrogen cyanamide has been used for many years in different fruit trees such as sweet cherry, peach, apricot, kiwifruit, and grapevine, its molecular mechanism of action remains unknown (Ionescu et al., 2017). It has been demonstrated *in vitro* that hydrogen cyanamide can be converted to hydrogen cyanide and nitroxyl by the action of catalase (Shirota et al., 1987).

Hydrogen cyanide has been implicated in seed germination (Zagórski and Lewak, 1983; Bogatek et al., 1991; Bethke et al., 2006; Oracz et al., 2009) and bud dormancy release (Tohbe et al., 1998). Hydrogen cyanide release has been measured in different reproductive tissues of *Eucalyptus cladocalyx* (F. Muell). The highest content was detectable in young buds, followed by older buds and flowers (Gleadow and Woodrow, 2000). Due to the cyanogenic nature of CNglcs, we hypothesize that they could be a source of hydrogen cyanide and thus inducers of endodormancy release. The aim of this study was therefore to investigate the possible role of CNglcs in endodormancy release of almond and sweet cherry.

## Materials and Methods

### Plant Material Sampling

#### Almond

Flower buds and different parts of the flower (pistils, petals, and sepals) of five different almond cultivars chosen by their differences in flowering time (very early: ‘Achaak’ and ‘Desmayo Largueta,’ early: ‘S3067,’ late: ‘Lauranne’ and extra-late: ‘Penta’) (**Table 1**) were collected every 2 weeks, from November 5th, 2013 to March 24th, 2014 (11 time points), in the experimental orchard of CEBAS-CSIC, in Santomera (Murcia, South-East Spain, 38.1095222, -1.037975). With the exception of the bitter cultivar ‘S3067’ (*sksk*), all used cultivars are sweet-kernelled, defined by the dominant *Sk (Sweet kernel)* gene locus (**Table 1**).

**Table 1:** List of cultivars classified by their flowering time and kernel bitterness (defined by *Sk* locus).

Cultivar	Flowering time	Genotype	Endodormancy release	CR (CU)	HR (GDH)
**Achaak**	Very early (25th January)	Sweet (*Sk*/–)	30th-November	231	9276
**Desmayo L.**	Very early (30th January)	Sweet (*Sksk*)	6th-December	306	9395
**S3067**	Early (13th February)	Bitter (*sksk*)	12th-December	391	11297
**Lauranne**	Late (28th February)	Sweet (*SkSk*)	25th-December	533	12399
**Penta**	Extra-late (15th March)	Sweet (*SkSk*)	02nd-February	819	7871


*Chilling (CR) and heat (HR) requirements for breaking dormancy and flowering time of the almond cultivars used in this study. Abbreviations: CU, chill units; GDH, growing degree hours.*

Four to six branches were collected per time point following the phenological stages A to F (Felipe, 1977), as previously described in (Sánchez-Pérez et al., 2010). Samples were snap-frozen in liquid nitrogen and kept at -80°C.

#### Sweet Cherry

**FIGURE 2:**
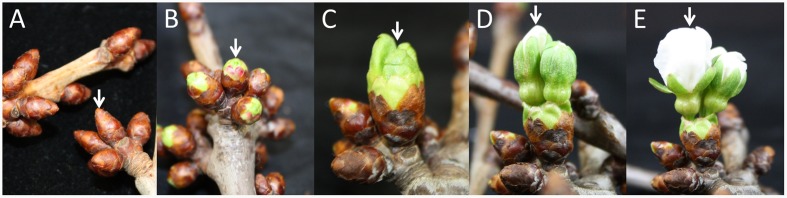
**Significant phenological stages of the sweet cherry flower buds sampled in this study.** **(A)** stage A, **(B)** stage BC, **(C)** stage D, **(D)** stage E. **(E)** stage F. White arrows indicate flower buds in the appropriate stages.

Flower bud samples were taken from sweet cherry trees of the cultivar ‘Burlat’ on ‘Santa Lucia’ rootstock, grown in the experimental orchard of the INRA Bordeaux in Toulenne (south-west France, 44.575503, -0.283008). ‘Burlat’ is considered a reference cultivar in sweet cherry. The chill requirements (Richardson et al., 1974) of ‘Burlat’ in Toulenne were calculated (976 CU in 2015, Bénédicte Wenden, personal communication) and when 709.5 CU were fulfilled – still in the endodormant state – 20 cm long branches were cut from the trees and placed at controlled conditions in a growth chamber (forcing conditions: 25°C day/20°C night, 16 h light/8 h dark (6–22:00), 30 μmol/m/s light intensity, direct lighting, 40% relative humidity). The branches were immersed in tap water, which was changed every 3 days. Flower buds [stage A–E according to Baggiolini (1952) (**Figure 2**)] were sampled up to 17 days after treatment, always between 9 and 12 am. Samples were snap-frozen in liquid nitrogen and kept at -80°C.

### Accumulation of Chill and Heat for Breaking Dormancy and Flowering

#### Almond

Three 40 cm long branches of each almond cultivar were collected every 2 weeks (**Table 2**) from the field and placed in a growth chamber in controlled conditions (light period of 16 h at 25°C, 40% relative humidity and darkness period of 8 h at 20°C and 60% relative humidity). The branches were placed in jars and immersed in a 5% saccharose and 1% aluminum sulfate solution, which was replaced every 5 days. The developmental stage of the flower buds was measured 10 days later, establishing the date of endodormancy release when 50% of the flower buds were in the BC stage. In the field, the flowering date was determined as the date where 50% of the flowers of the tree had fully opened (F stage).

**Table 2:** Sampling time points for the almond and sweet cherry samples used in this study.

Almond				Cherry		

Batch no	Date	CU	GDH	Day of sampling	Date	Stages
1	5th of November	0	0	0	19.01.15	A
2	18th of November	56	512	1	20.01.15	A
3	2nd of December	260	2244	3	22.01.15	A
4	16th of December	441	4243	7	26.01.15	B,C
5	30th of December	590	6455	10	29.01.15	B,C,D
6	13th of January	673	9151	15	02.02.15	B,C,D,E
7	27th of January	754	11824	17	05.02.15	B,C,D,E
8	10th of February	859	14338			
9	24th of February	902	17272			
10	10th of March	917	20339			


*Almond samples were taken in the field from November to March and Chill Units (CU) and heat requirements (GDH) indicated for each batch. For cherry, the sampling was conducted in a growth chamber at seven different time points from January to February.*

Calculation of chill requirements was performed in Chill Units (CU) according to the method of Richardson et al. (1974), as a function of the number of hours at a certain temperature range accumulated from November 15th. This method takes into account that temperatures outside this range counteract chill accumulation (chill negation) (Erez et al., 1979).

Heat requirements were calculated as growing degree hours (GDH), which is the hourly temperature minus 4.5°C. The heat requirements of each cultivar were calculated as the number of GDH accumulated between the release of endodormancy and flowering time, when 50% of flowers were open (F50) (**Table 1**).

#### Sweet Cherry

Starting in November 2014, the endodormancy status of ‘Burlat’ flower buds was determined. At each time point, three branches were cut from the trees and placed in a growth chamber in controlled conditions. Bud break was measured as the percentage of flower buds that pass developmental stage BC (Baggiolini, 1952) (**Figure 2**). With 50% of all flower buds beyond stage C, endodormancy was considered broken. In this experiment, it was not possible to determine flowering time (50% of flowers open), because only 18% of all flower buds opened to the point of a full flower. The reason for this might be a lack of nutrient resources in the branches.

### LC-MS Analysis

Cyanogenic glucosides were analyzed as described previously (Pičmanová et al., 2015). Samples (100 mg) were ground to a fine powder in liquid nitrogen, mixed with 400 μL 85% methanol, boiled 5 min, placed on ice and centrifuged (5 min, 20,000 × *g*). Aliquots (20 μL) of the supernatant were mixed with 70 μL of water and 10 μL of 500 μM internal standard (linamarin) and filtered through a filter plate (0.45 μm, Millipore) by centrifugation (5 min, 1,107 × *g*).

LC–MS/MS was carried out using an Agilent 1100 Series LC (Agilent Technologies) coupled to a Bruker HCT-Ultra ion trap mass spectrometer (Bruker Daltonics). A Zorbax SB-C18 column (Agilent; 1.8 μm, 2.1 mm × 50 mm) maintained at 35°C was used for separation. The mobile phases were: (A) water with 0.1% (v/v) HCOOH and 50 mM NaCl; (B) acetonitrile with 0.1% (v/v) HCOOH. The gradient program was: 0–0.5 min, isocratic 2% B; 0.5–7.5 min, linear gradient 2–40% B; 7.5–8.5 min, linear gradient 40–90% B; 8.5–11.5 min isocratic 90% B; 11.6–17 min, isocratic 2% B. The flow rate was 0.2 ml⋅min^-1^ but increased to 0.3 ml⋅min^-1^ in the interval 11.2–13.5 min. ESI–MS^2^ was run in positive mode. The data was analyzed using the Bruker Daltonics programme Data Analysis 4.0. Extracted ion chromatograms for specific [M+Na]^+^ adduct ions (as NaCl is added to one of the mobile phases, the great majority of adducts formed are [M+Na]^+^; we could also see [M+H]^+^ and [M+NH_4_]^+^, but these are minute in comparison with the sodium adducts) and their MS^2^ profiles were used to identify the compounds.

**Table 3** shows the names, structures, and retention times of all the compounds detected in this study. Amygdalin was bought from Sigma–Aldrich. Prunasin was chemically synthesized (Møller et al., 2016). Prunasin amide, prunasin acid, prunasin anitrile, 1-*O*-benzoyl-β-D-glucopyranose, prunasin-6′-β-D-apioside and prunasin-6′-β-D-xyloside were chemically synthesized (Motawia MS, unpublished work). The reference compounds were used for absolute quantification in a range of concentrations from 0.5 to 125 μM. As for the relative quantifications presented, the ionization efficiency of prunasin and its derivatives may differ by a factor of approximately two, and hence the ratios expressed as percentages of prunasin content are correct within this span (Pičmanová et al., 2015). The MS and MS^2^ spectra observed for each compound were in agreement with the spectra previously reported (Pičmanová et al., 2015).

**Table 3:** Structures of compounds detected in this study.

Compound	Chemical name	*m/z* [M+Na]^+^	r.t. [min]	
Prunasin	(*2R*)-2-(β-D-Glucopyranosyloxy)phenylacetonitrile	318	7	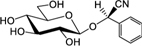

Prunasin amide	(*2R*)-2-(β-D-Glucopyranosyloxy)phenylacetamide	336	4.4	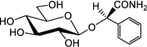

Prunasin acid	(*2R*)-2-(β-D-Glucopyranosyloxy)phenylacetic acid	337	5.7	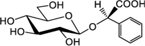

Prunasin anitrile	Benzyl β-D-glucopyranoside	293	6.5	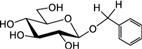

Prunasin pentoside	(*2R*)-2-(Pentosyl(1→6)-β-D-glucopyranosyloxy)phenylacetonitrile	450	6.9	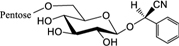

Prunasin anitrile apioside	Benzyl β-D-apiofuranosyl-(1→6)-*β*-D-glucopyranoside	425	6.8	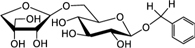

Prunasin anitrile xyloside	Benzyl β-D-xylopyranosyl-(1→6)-β-D-glucopyranoside	425	6.9	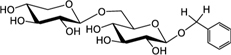

Prunasin anitrile arabinoside	Benzyl *a*-L-arabinopyranosyl-(1→6)-β-D-glucopyranoside	425	6.7	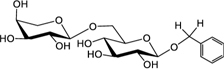

Amygdalin	(*2R*)-2-[*β*-D-glucopyranosyl-(1→6)-*β*-D-glucopyranosyloxy]phenylacetonitrile	480	6.6	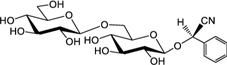

β-D-Glucose-1-benzoate	1-*O*-Benzoyl-β-D-glucopyranose	307	6.7	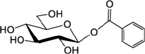


**m/z*, mass-to-charge ratio; r.t., retention time.*

Samples were assayed in two to three technical replicates, except for the last time point of the prunasin content in S3067.

### qRT-PCR Analysis in Sweet Cherry

Quantitative real-time polymerase chain reaction (qRT-PCR) based expression analysis was carried out on 12 selected genes using three reference genes (*TEF2*, *18s rRNA*, and *RPL13)* (**Table 4**). The targeted gene sequences were based on homologous genes derived from different *Prunus* species and preliminary transcriptomic data from sweet cherry ‘Burlat’ flower buds (Ionescu et al., 2017).

**Table 4:** Primer sequences for qRT-PCR analysis of reference and target genes.

Gene	Accession number	Forward primer (5′–3′)	Reverse primer (5′–3′)	Amplicon size (bp)	C %	I %
*18s rRNA*	–	GTGAGGCCATATGCAGTGAAG	TAACGTCCTCTGGCTGTGAAG	133	72	85
*RPL13*	–	GAGGAGCTTGCCAATGCTAC	CTCGCACCAACATGACGTTC	161	78	68
*TEF2*	–	GGGAGATGATGTCGTCTGAT	TTGTCCTCAAACTCGGATAGT	121	75	89
*Catalase*	EF165590.1	GCATTTGTTGTCCCTGGTATC	TCACTGGGAGCTGCATATAG	118	76	92
*Peroxidase*	–	CAGCTCAATTCCATGTTTGC	GACTGAAGCTGTAAATCCGA	124	73	94
*CYP79D16*	AB920488.1	CGGCCATGAGAAGATCATAAAG	AGTCTACTGGGACCTTGTTTC	119	59	87
*CYP79A68*	XM 008243186.1	GCAAACCACGGAGCTG	CCCACTACCCTATCTAGTTCC	129	75	66
*CYP71AN24*	AB920492.1	GGGAAGCAATGTCTGATGTAAA	CTCAAACCTCTCTGGCATAAAC	137	83	76
*CYP71AP13*	XM_008241135.1	TCAAGGCTATCATCTTGGACA	AACACCTCGTACTTCTGCTT	131	71	93
*AH1*	U26025.2	CATTCACTGTGCTTCTCTCAAC	CTTGGTCCTCTACCATCTTCTT	123	81	82
*PH5*	XM_008245363.1	CAATGAAGGAGGGTGCTAATG	AGTGCGTCGATAGTTTTGAG	150	83	84
*ACC oxidase*	NM 001293254.1	CTTCCCAATCATCAACTTGGA	CCATGACTCACAAGCTCAAA	111	77	80
*ACC synthase*	NM 001293270.1	CTCTCCTTACTATCCAGCATTTT	TGATGTTGTTCTTTTGGGCT	149	74	91
*SAM synthetase*	JX876836.1	GTGTCCACACTGTCCTAATTTC	CAAGGTACTTCTCAGGGATCA	114	75	86
*CAS*	XM 008246435.1	ACTCATCGGTAGAACTCCCA	AAGTGCTGGTCTGTCTTTGA	121	72	72


*Genes are displayed with their corresponding accession number used for primer design. Herein, sequence data that derived from preliminary transcriptomic data (Ionescu et al., 2017) is indicated with a hyphen and shown in SD.1. PCR products generated on sweet cheery ‘Burlat’ flower bud cDNA were sequenced and sequence coverage (C) as well as identity (I) in percent were assessed.*

Sweet cherry ‘Burlat’ flower buds samples were obtained from branches kept for 1, 3, 7, 10, 15, and 17 days at controlled conditions. Frozen plant material was ground with mortar and pestle in liquid nitrogen. For each sample, total RNA was extracted using the Spectrum^TM^ Plant Total RNA Kit (Sigma–Aldrich, St. Louis, MO, USA) and 500 ng of RNA was used to generate cDNA using the iScript^TM^ cDNA Synthesis Kit (Bio-Rad, Hercules, CA, USA). Gene-specific primer pairs were designed for target and reference genes using two web based tools: NCBI’s Primer-BLAST^1^ and IDT’s^2^ PrimerQuest^©^ (**Table 4**). Primer efficiencies were 82 ± 12% and their sequence specificity was determined by sequencing the amplicon and comparing it to the original coding sequence used for initial primer design (see Supplementary Data Sheet 1, DS1). Obtained sequences were aligned to the associated coding sequences using a local alignment with Needleman–Wunsch algorithm (Needleman and Wunsch, 1970). Herein, sequence coverage was 75 ± 6% and identity was 83 ± 9%.

qRT-PCR was performed using a CFX384^TM^ real-time PCR detection system. Reactions were conducted in 8 μl volume using the DyNAmo Flash SYBR Green qPCR Kit (Thermo Fisher Scientific, Waltham, MA, USA) with each reaction containing 1x DyNAmo Flash SYBR Green qPCR Mix (2x), 5 ng of cDNA template and 625 nM of both forward and reverse primer. The following PCR protocol was used: 7 min at 95°C, [10 s at 95°C, 30 s at 60°C, 1x plate read] × 40 cycles, 1 min at 60°C. A melting curve was performed for each reaction. Further, no template controls as well as no RT controls were included. A standard curve for TEF2 was used as interrun control using the deduced PCR efficiency as factor for interrun deviation. Relative gene expression levels were computed from the qPCR data using the ΔΔCq calculation method (Livak and Schmittgen, 2001). Therein a normalization factor based on the expressional variation of three reference genes among the examined samples was used. This factor was obtained using geNorm version 3.5 (Vandesompele et al., 2002).

## Results and Discussion

### Prunasin and Amygdalin in Flower Buds of Almond and Sweet Cherry

**FIGURE 3:**
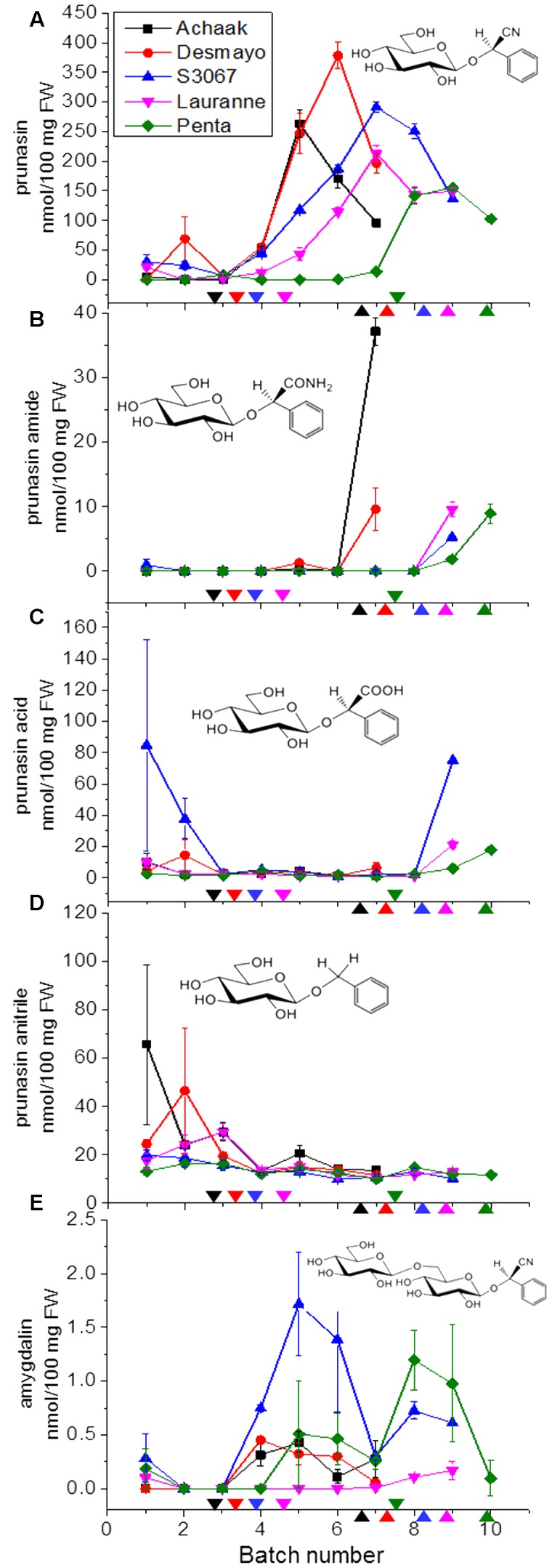
**Prunasin** **(A)**, prunasin amide **(B)**, prunasin acid **(C)**, prunasin anitrile **(D),** and amygdalin **(E)** content in flower buds from five almond cultivars with different flowering times (earliest to latest: Achaak, Desmayo, S3067, Lauranne and Penta) from the 5th of November to 24th March. Downward arrows indicate endodormancy release and upward arrows indicate flowering time. Bars indicate standard error.

**FIGURE 4:**
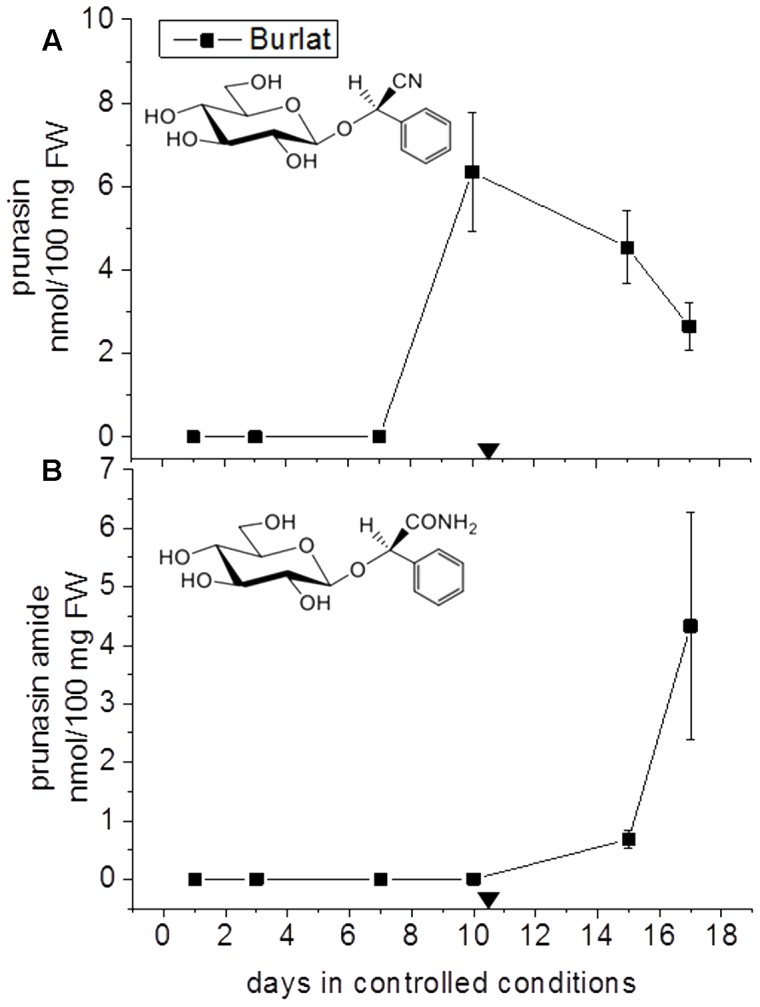
**Prunasin** **(A)** and prunasin amide **(B)** content flower buds of the sweet cherry cultivar ‘Burlat’. Downward arrows indicate endodormancy release (10.4 days). Data points represent three biological replicates. Bars indicate standard error.

The CNglc prunasin (**Table 3**) was detected in all five almond cultivars during the entire developmental period of the buds from dormancy to flowering (**Figure 3A**). Prunasin was also detected under controlled conditions in flower buds of the sweet cherry ‘Burlat,’ but in levels approxiamtely 10-fold lower than in almond (**Figure 4A**). Amygdalin, the other CNglc present in almond, was detected in all five cultivars in minute amounts, approximately 200-fold lower, compared to prunasin (**Figure 3E**). This is within the range (37–300-fold lower) that had been previously observed in two almond cultivars (Ramillete-sweet and S3067-bitter), when prunasin and amygdalin were measured in the leaves of almond trees after the almonds had been harvested (Figure 5 in Sánchez-Pérez et al., 2008). In sweet cherry flower buds, no amygdalin was detected. The di-glucoside amygdalin is present in very minute amounts compared to the monoglucoside prunasin and this is in agreement with a previous observation (Frehner et al., 1990; Dicenta et al., 2002; Sánchez-Pérez et al., 2008). The situation is reverse in bitter almond seeds where amygdalin is the dominating cyanogenic glucoside. In vegetative parts of the tree, prunasin is always the dominating cyanogenic glucoside present.

In general, during the entire dormancy-flowering period, the level of prunasin was highest in the early cultivars Achaak and Desmayo, followed by S3067. Lauranne and Penta contained the smallest amounts of prunasin (**Figure 3A**). The prunasin profiles obtained shared clear relations to the dates of dormancy breaking and flowering time. In all five almond cultivars as well as in the single sweet cherry cultivar, prunasin started to accumulate at the time of dormancy release or shortly thereafter and reached its maximum just before flowering took place. This may suggest that prunasin plays a role in flower development after dormancy is broken.

Dissection of almond flowers enabled detection of prunasin and minute amounts of amygdalin in pistils, petals and sepals of all five almond varieties (**Supplementary Figure S1**). S3067 was the only variety where prunasin could be detected in the pollen, but the amount of pollen available was too low to acquire biological and technical replicates. In relation to this, amygdalin content has previously been reported in almond pollen at about 1890 ppm (London-Shafir et al., 2003) and reported to deter inefficient pollinators, thus allowing more efficient pollination by honeybees, adapted to tolerate higher levels of amygdalin. Prunasin had previously been reported detected in sepals, petals, pistils, and pollen of flowers from bitter and sweet almond cultivars (Abarrategui, 2010). Amygdalin levels were almost zero, except in the bitter cultivars.

In the case of *Lotus japonicus*, the two aliphatic CNglcs linamarin and lotaustralin are present throughout in the flower tissue (Lai et al., 2015). As mentioned previously (**Figure 1B**), bioactivation of the CNglc takes place only when specific β-glucosidases come into contact with their corresponding substrate. In *L. japonicus* the reproductive organs are only cyanogenic when a specific β-glucosidase BGD3 is expressed (Lai et al., 2015). Hydrogen cyanide release was derived specifically from the keel and enclosed reproductive organs of the flower. Sepals, wings, buds, and pods also contained the cyanogenic glucosides linamarin and lotaustralin, but no release of any hydrogen cyanide from these tissues was observed because the β-glucosidases were not present in these tissues (Lai et al., 2015). It needs to be investigated whether or not a particular β-glucosidase might also be expressed in almond and cherry flower buds.

The presence of CNglcs in flowers of other species has previously been reported. Within the *Prunus* genus, prunasin was quantified in flowers of *P. avium*, whereas amygdalin was not detected (Nahrstedt, 1972). Prunasin as well as amygdalin were identified in flowers of *P. yedoensis* Matsum (Matsuoka et al., 2011). Five different CNglcs were also found in flower buds of *Eucalyptus camphora* subsp. *humeana*, namely prunasin and the diglucosides amygdalin and eucalyptosins A, B, and C (Neilson et al., 2011). In *Turnera ulmifolia* L., the content of CNglcs decreased to zero when the plant began to flower (Schappert and Shore, 2000) indicating complete endogenous turn-over of CNglcs for alternative uses. CNglcs have also been detected in flower tissues of *Grevillea* species, *Linum usitatissimum* L. (flax), *L. japonicus* L., *Ryparosa kurrangii* B.L. Webber (rainforest tree) and *E. camphora* L.A.S. Johnson and K.D. Hill (Lamont, 1993; Niedźwiedź-Siegieñ,, 1998; Forslund et al., 2004; Webber and Woodrow, 2008; Neilson et al., 2011).

### Putative Derivatives of Prunasin in Flower Buds

**FIGURE 5:**
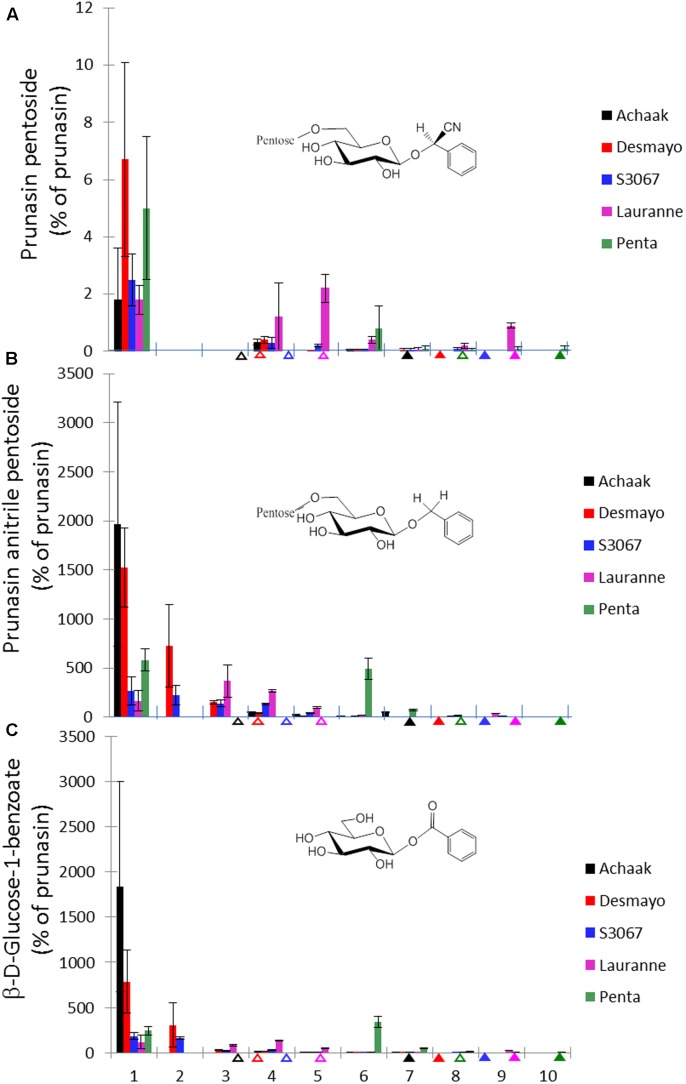
**Prunasin pentoside** **(A)**, prunasin anitrile pentoside **(B)** and β-D-glucose-1-benzoate **(C)** (% of prunasin) in flower buds from five almond cultivars with different flowering times (earliest to latest: Achaak, Desmayo, S3067, Lauranne and Penta) from the 5th of November to 24th March. Upward empty arrows indicate endodormancy release and upward filled arrows indicate flowering time. Bars indicate standard error.

**FIGURE 6:**
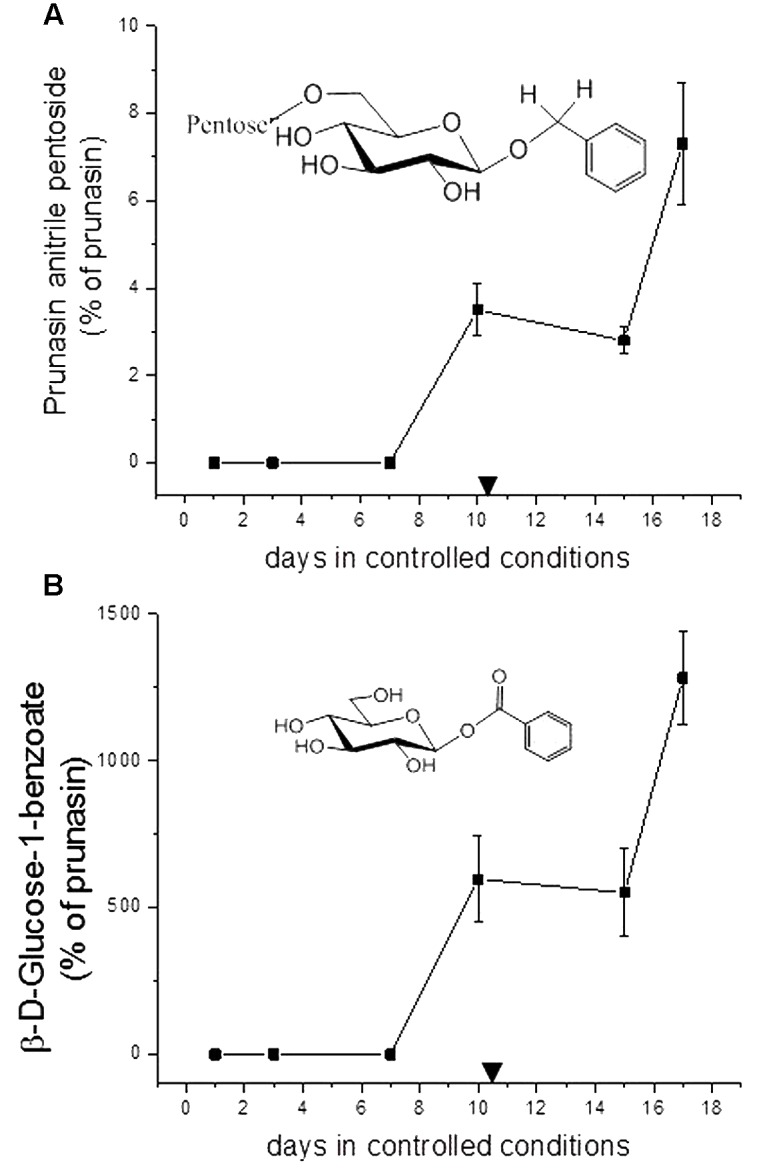
**Prunasin anitrile pentoside** **(A)** and β-D-glucose-1-benzoate **(B)** (% of prunasin) in sweet cherry ‘Burlat’ flower buds. Downward arrows indicate endodormancy release (10.4 days). Data points represent three biological replicates. Bars indicate standard error.

In addition to prunasin and amygdalin, structurally related derivatives were also found in the flower buds of the five almond cultivars (**Table 3** and **Figures 3B–D**, **5**), in almond pistils, sepals and petals (**Supplementary Figure S1**) and in the one sweet cherry cultivar analyzed in this study (**Table 3** and **Figures 4B**, **6**). The prunasin derivatives prunasin amide, prunasin acid, prunasin anitrile, and the diglycoside prunasin pentosides were all present in amounts much lower than prunasin. In contrast, the non-cyanogenic diglycoside prunasin anitrile pentosides (prunasin anitrile arabinoside and xyloside in almond and most probable prunasin anitrile apioside in cherry) were highly abundant at certain stages of flower development (**Figures 5**, **6**).

The content of prunasin amide (**Figures 3B**, **4B**) displayed a very interesting and consistent pattern in the five almond and the single cherry cultivar analyzed. Prunasin amide was not detectable until it peaked very close to flowering time. In almond, the highest amount of prunasin amide was found in the earliest cultivar (Achaak). In all studied cultivars, the peak of prunasin amide coincided with a decrease in prunasin levels, indicating turnover of prunasin into its amide. The conversion of prunasin to prunasin amide may occur non-enzymatically via the Radziszewski reaction in the presence of hydrogen peroxide (Sendker et al., 2016). Hydrogen peroxide is produced during flower development (Kuroda et al., 2002). Although present in small amounts, formation of prunasin amide may thus serve as a quenching reaction to avoid toxic hydrogen peroxide levels (Møller, 2010). Alternatively, prunasin amide might be formed from prunasin catalyzed by a bifunctional nitrilase or by a nitrile hydratase (Pičmanová et al., 2015).

**FIGURE 7:**
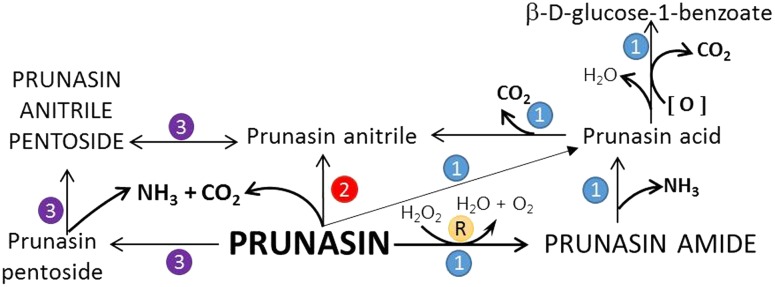
**Proposed turnover pathways (1, 2, 3, and R) for prunasin without the release of hydrogen cyanide (after Pičmanová et al., 2015; Sendker et al., 2016).** In pathway 1, prunasin is sequentially converted into its amide and/or acid and anitrile; moreover, β-D-glucose-1-benzoate may be formed from prunasin acid. Prunasin anitrile might also be produced directly from prunasin without intermediates (pathway 2). Pathway 3 entails the glycosylation of prunasin to prunasin pentoside and its further conversion into prunasin anitrile; a glycosyltransferase and a β-glycosidase are involved in these processes. R is the Radziszewski reaction where, by addition of hydrogen peroxide, prunasin amide can be formed, liberating water and oxygen, what may quench the ROS produced during dormancy release. Font size represent the abundance of the compounds in the samples analyzed.

The presence of prunasin acid (**Figure 3C**) was detected at the beginning of almond flower bud development, although with relatively high standard error margins. In the mid-late cultivars S3067, Lauranne and Penta, small amounts of prunasin acid were observed to accumulate at the time point of flowering. Prunasin acid is likely formed from the prunasin amide (**Figure 7**). The levels of prunasin acid in the cultures Achaak and S3067 were close to zero. Low amounts of prunasin anitrile were accumulated in the almond cultivars, with peak levels before endodormancy release (**Figure 3D**).

In addition to the monoglucosides described above, two diglycosides (pentosides) derived from prunasin were identified in this study. Absolute quantification was not possible due to the lack of reference compounds. Therefore, we expressed the levels of these compounds as % of prunasin (**Figures 5**, **6**). The levels of prunasin pentoside in almond (potentially a mixture of two prunasin pentosides) (Pičmanová et al., 2015) were higher at the beginning in the dormant stage, where CU had not yet accumulated (**Figure 5A**). All almond cultivars exhibited the presence of prunasin anitrile pentoside during endodormancy release, reaching relative amounts of up to 2000% of prunasin (e.g., Achaak, **Figure 5B**). In cherry, the levels of prunasin anitrile apioside increased toward the end of the experiment (**Figure 6A**).

In senescent leaves of *P. laurocerasus* L., novel benzoic acid esters have recently been reported as formed from prunasin (Sendker et al., 2016). This inspired us to investigate the possible presence of benzoic acid derivatives in almond and sweet cherry flower buds. A compound identified as β-D-glucose-1-benzoate was indeed found to be present in high amounts compared to prunasin in the flower buds of all studied almond cultivars as well as in the cherry cultivar (**Figures 5C**, **6B**). β-D-Glucose-1-benzoate was suggested to be formed as a novel extension of the oxidative catabolism of prunasin (Sendker et al., 2016). The amount of accumulated β-D-glucose-1-benzoate is high compared to the prunasin level implying that β-D-glucose-1-benzoate might also be synthesized by a different route in the flower buds. Moreover, in almond and cherry flower buds, the formation of β-D-glucose-1-benzoate from the corresponding aldehyde could potentially be connected to the release of hydrogen peroxide during dormancy release. As mentioned previously, hydrogen peroxide has been implicated in flower development in Japanese pear (*Pyrus pyrifolia* Nakai) (Kuroda et al., 2002).

These results are in accordance with a recent study reporting the presence and structural identification of CNglc derived metabolites including di- and tri-glycosides in cassava, sorghum, and almond (Pičmanová et al., 2015). The amides, acids and anitriles derived from prunasin and amygdalin were identified in seedlings of the bitter almond cultivar S3067. The levels of the derivatives of prunasin and amygdalin were generally much lower than those of their mother compounds. Prunasin amide, acid and anitrile were found in low levels in seeds, roots, shoots, and leaves of the seedling and at different stages of germination. Prunasin acid was the most abundant derivative in seeds, shoots, and leaves and prunasin anitrile was most abundant in roots. An important increment of the prunasin derivatives was observed in the seed at the beginning of the germination (Pičmanová et al., 2015). Similarly, minor components related to CNglcs were detected in *P. persica* seeds: amygdalin acid, prunasin acid, benzyl gentiobioside and benzyl glucoside (Fukuda et al., 2003). The latter two compounds correspond to the amygdalin anitrile and prunasin anitrile compounds denoted in our study.

Our current study provides further evidence in support of the conclusions by Pičmanová et al. (2015) that CNglcs occur together with their putative structural derivatives: amides, acids and anitriles. In this respect, it was suggested that these derivatives could play a role in the recycling of reduced nitrogen. An alternative endogenous turnover pathway was proposed in which CNglcs are converted to non-CNglcs, without release of HCN (**Figure 1D**). Hypothetically, amides, acids, and anitriles are produced from CNglcs in this turnover pathway, with a concomitant release of NH_3_ and CO_2_. In this form, reduced nitrogen and carbon originating from the CNglcs could be utilized in primary metabolism. This alternative pathway might operate concurrently with the “conventional” bioactivation pathway, in which amygdalin and prunasin are hydrolyzed and decomposed into benzaldehyde and HCN; the latter is further detoxified through β-cyanoalanine into asparagine, aspartate and NH_3_.

Based on the general alternative turnover pathway proposed by Pičmanová et al. (2015), we suggest three possible routes for the turnover of CNglcs in *Prunus* species (**Figure 7**), starting with the hydrolysis of amygdalin to prunasin. Then, in the first route, prunasin is further hydrolysed to prunasin amide and/or acid and NH_3_. Prunasin acid is converted into prunasin anitrile or to β-D-glucose-1-benzoate, with a release of CO_2_. In the second route, prunasin is converted directly into the corresponding anitrile with the release of NH_3_ and CO_2_. NH_3_ as CO_2_ produced in these proposed pathways may be channeled into primary metabolism. In a third route, prunasin is glycosylated to a prunasin pentoside that would also produce NH_3_ and CO_2_, when converted to prunasin anitrile pentoside. The latter could also be deglycosylated into prunasin anitrile.

### Other Functions of Cyanogenic Glucosides

Cyanogenic glucosides are biosynthesized from amino acids, therefore the plant must mobilize and transport these precursor substances to the sites where CNglcs are needed. Supply of nitrogen for the biosynthesis of CNglcs is especially important in young tissues, which are weaker than mature tissues and are in greater need of defense against pathogens and herbivores. On the other hand, at times where defense responses are less urgent, the plant can reuse nitrogen from CNglcs and redirect it into primary metabolism (Vries et al., 2017).

In Eucalyptus, it has been demonstrated that up to 20% of leaf nitrogen is stored in CNglcs, with the highest levels in young and reproductive tissues (Gleadow and Woodrow, 2000). In spring, coinciding with the flowering period, there was an important allocation of nitrogen to the reproductive tissues in detriment to the leaves to form CNglcs. The levels of these compounds decreased gradually during fruit development (buds – flowers – fruits).

Cyanogenic diglycosides may have additional functions as transport forms, pollinator attractants and germination inducers. In *E. camphora* trees, the highest levels of diglucosides were found in flower buds and expanded leaves (Neilson et al., 2011). Theoretically, the diglucosides are synthesized in the expanded leaves and then transported to the developing flower buds. The levels of cyanogenic diglucosides were much lower in immature fruits suggesting that nitrogen was remobilized and used during the flower development (Neilson et al., 2011).

### HCN Factor

As previously mentioned, HCN may be produced and metabolized during flower bud development, indicated by a decrease in CNglc levels. Past as well as recent studies have also shown that HCN may activate the flower bud and the flower opening in *Lemna paucicostata* and grapevine (Tanaka et al., 1983; Tohbe et al., 1998).

Interestingly, HCN has also been reported in releasing seed dormancy in orthodox seeds (Roberts, 1973; Roberts and Smith, 1977) by inducing the formation of Reactive Oxygen Species (ROS); ROS in turn activates a cascade involving Ethylene Response Factor 1 (ERF1), which leads to the production of germination-associated proteins (Oracz et al., 2009). Extensive literature describes the importance of the HCN in seed germination. Considering the common mechanisms regulating seed and bud dormancy, this process could be similar in endodormancy release (Taylorson and Hendricks, 1973; Bogatek et al., 1991; Flematti et al., 2013).

### Involvement of Cyanogenic Glycosides in Regulation of Sweet Cherry Flower Bud Dormancy as Monitored by qRT-PCR Analysis

**FIGURE 8:**
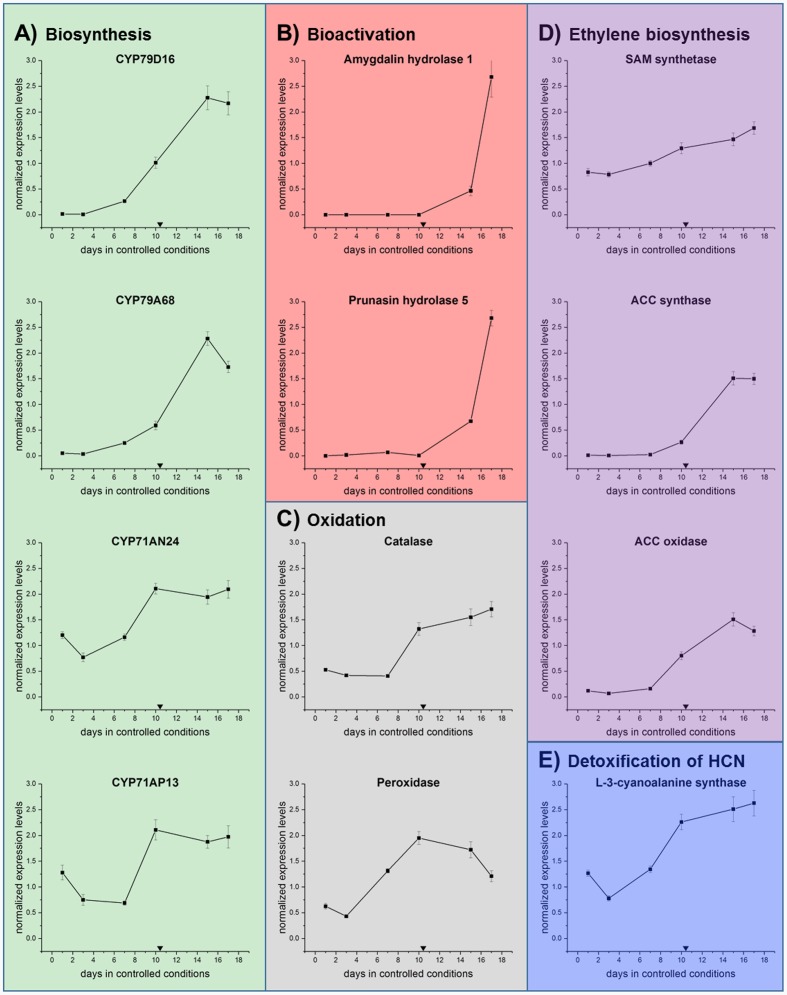
**qRT-PCR analysis in the first genes involved in the** **(A)** biosynthesis of CNglcs (CYP79D16, CYP79A68, CYP71AN24, CYP71AP13), **(B)** bioactivation (*Amygdalin hydrolase* and *prunasin hydrolase*), **(C)** oxidation (*catalase* and *peroxidase*), **(D)** ethylene biosynthesis (*SAM synthase, ACC synthase*, and *ACC oxidase*) and **(E)** detoxification (*L-3-cyanoalanine synthase*) in sweet cherry ‘Burlat’ flower bud samples under controlled conditions. Downward arrows indicate endodormancy release (10.4 days). Data points represent one biological replicate analyzed in three technical replicates.

To obtain more information on the possible regulation of these processes, qRT-PCR analysis was performed on the sweet cherry samples. The expression levels of a selected number of genes were analyzed (**Figure 8**). In CNglcs biosynthesis: CYP79 and CYP71 (**Figure 8A**). In bioactivation: *amygdalin* and *prunasin hydrolase* (**Figure 8B**). In oxidation reactions: *catalase* and *peroxidase* (**Figure 8C**). In ethylene biosynthesis: *SAM synthase*, *ACC synthase*, and *ACC oxidase* (**Figure 8D**). In the detoxification pathway: *L-3-cyanoalanine synthase* (**Figure 8E**).

Both CYP79 genes displayed their highest level of expression after dormancy release, indicating that CNglcs biosynthesis takes place during early flower development in sweet cherry (**Figure 8A**). In buds of Japanese apricot (*P. mume*), CYP79A68 was the only examined cytochrome P450 monooxygenase encoding gene showing a substantial level of expression (Yamaguchi et al., 2014). Further Yamaguchi et al. (2014) reported that CYP79D16, but not CYP79A68, catalyzed the conversion of L-phenylalanine into *E*-phenylacetaldoxime. The second step in CNglcs biosynthesis is mediated by CYP71s (Sánchez-Pérez et al., 2008), such as CYP71AN24 and CYP71AP13 (Yamaguchi et al., 2014). In general, the expression of the two CYP71 encoding genes was transiently down-regulated shortly before dormancy release and subsequently increased again (**Figure 8A**). This is in accordance with the results for the CYP79s. Further CYP71AN24, but not CYP71AP13, catalyzed the conversion of *E*-phenylacetaldoxime into mandelonitrile (Yamaguchi et al., 2014). Hence, future studies have to reveal the functional properties and substrate specificities of CYP79s and CYP71s in sweet cherry to resolve the biosynthesis of prunasin in sweet cherry.

As previously mentioned, the degradation of CNglcs is initiated by β-glycosidases, in Prunus species called amygdalin hydrolase (AH) and prunasin hdyrolase (PH). *Ah1* and *Ph5* (Zhou et al., 2002) were examined in this study (**Table 4**) as they were the most similar characterized hydrolases between *P. serotina* and *P. dulcis* (Sánchez-Pérez et al., 2012). As shown in **Figure 8B**, both genes display transcriptional activity solely after dormancy release. In the case of *Ph5*, this fits well with the decrease of prunasin levels at around the same time point, indicating its degradation.

L-3-Cyanoalanine synthase (CAS) activity serves as an indicator for HCN release because of its essential involvement in HCN detoxification (Floss et al., 1965). After a transient peak, *CAS* transcription decreased and rised again during dormancy release and during flower development (**Figure 8E**).

### Involvement of Oxidative Stress Regulating Factors in Sweet Cherry Bud Dormancy Release as Monitored by qRT-PCR Analysis

Pathways involved in oxidative stress regulation have previously been shown to be active during dormancy release in several different perennials (Horvath, 2009; Cooke et al., 2012). In our study, catalase expression decreased slightly and then increased again just before dormancy was released (**Figure 8C**). Several studies found catalase activity to be affected by both natural and artificially induced bud break (Nir et al., 1986; Pérez and Lira, 2005; Amberger, 2013). Catalases are known to catalyze the conversion of H_2_O_2_ to water and oxygen (Chelikani et al., 2004). Thus, the inhibition of catalase gene transcription and enzyme activity by, e.g., HCN released from the cyanogenic glucoside hydrolysis could result in increased hydrogen peroxide levels. In this study, the subsequent up-regulation of the catalase gene after dormancy release might decrease H_2_O_2_ levels again, which is consistent with a steady decrease in H_2_O_2_ content after dormancy release found in flower buds of *P. pyrifolia* (Japanese pear) (Kuroda et al., 2002).

In addition to catalase, a range of peroxidases are able to reduce H_2_O_2_ to water and have been shown to be induced in response to oxidative stress during dormancy release in grape buds (Veitch, 2004; Keilin et al., 2007). The peroxidase gene examined in our study (**Figure 8C**) was most highly expressed at bud dormancy release, indicating that peroxidase functions mainly during the transition from dormancy to flowering in sweet cherry, which is similar to results acquired in Japanese pear (Bai et al., 2013). Differently regulated peroxidases during transition of dormancy release were observed in prior studies. For instance, in buds of Chinese cherry (*P. pseudocerasus* Lindl.), different peroxidase encoding genes were either down-regulated before, during and after dormancy release under natural conditions (Zhu et al., 2015). This suggests a pattern of alternating activities among a set of peroxidases that regulate oxidative stress during bud dormancy release. Peroxidases were found to be up-regulated in buds of peach and leafy spurge (*Euphorbia esula* L.) (Jia et al., 2006; Leida et al., 2010) and down-regulated in grapevine in regard to dormancy release (Pacey-Miller et al., 2003). The examined peroxidase gene in our study was down-regulated before dormancy release, which coincides with our observation of a decreased catalase expression, potentially giving rise to a transient increase in ROS. Subsequently enhanced expression of peroxidase and catalase encoding genes during and after bud dormancy release might then cooperatively reduce oxidative stress.

### Involvement of Ethylene Regulation in Sweet Cherry Bud Dormancy Release as Monitored by qRT-PCR Analysis

Transcript analysis of three key genes encoding enzyme involved in ethylene biosynthesis, namely *S-adenosyl-methionine (SAM) synthetase*, *1-aminocyclopropane-1-carboxylic acid (ACC) synthase* and *ACC oxidase* were conducted and demonstrated that *ACC synthase* and *ACC oxidase* were initially expressed shortly before dormancy release (**Figure 8D**). Those results suggest that ethylene biosynthesis was initiated before dormancy release in sweet cherry. In grapevine, the effect of different temperatures and sampling dates on bud break and ACC content was studied, seeing that under low temperatures, bud break was associated with the promotion of ethylene biosynthesis (El-Shereif et al., 2005). Heat shock experiments demonstrated that ACC and ethylene accumulated toward dormancy release in grapevine (Tohbe et al., 1998). Transcription of the gene encoding *ACC synthase* was induced in flower buds in Japanese pear (Bai et al., 2013). Exogenous application of ACC has been reported to enhance dormancy release. The same effect was not observed upon exposure to ethylene (Iwasaki, 1980). Since hydrogen cyanide is formed in stoichiometric amounts with ethylene in the ACC oxidase catalyzed conversion of ACC, hydrogen cyanide is thought to be responsible for bud break in grapevine.

## Conclusion

Based on the results presented in this paper, two possible mechanisms for the involvement of CNglcs in bud break and flower development are proposed: (1) Turnover of CNglcs to their corresponding amides, acids and anitriles can recover reduced nitrogen and carbon dioxide, which may be utilized during these metabolically demanding physiological changes; (2) Prunasin and a number of endogenous turn-over products as well as formation of hydrogen cyanide from prunasin act as regulators of flower bud dormancy release and flowering time.

## Author Contributions

II and JD designed and conducted the main experiments and wrote the manuscript. MP conducted LC-MS data analysis and contributed to the manuscript. OG performed the qRT-PCR experiments and wrote the manuscript. MM synthesized most of the reference compounds and contributed to the manuscript. CO conducted the LC-MS analysis. JD assisted with the sweet cherry experiments. FD conducted almond flower bud sampling and the evaluation of the flower bud development. BM designed experiments and wrote the manuscript. RS-P designed and coordinated experiments, conducted LC-MS data analysis and wrote the manuscript.

## Conflict of Interest Statement

The authors declare that the research was conducted in the absence of any commercial or financial relationships that could be construed as a potential conflict of interest. The reviewer JZ and handling Editor declared their shared affiliation, and the handling Editor states that the process nevertheless met the standards of a fair and objective review.

## Footnotes

1 http://www.ncbi.nlm.nih.gov/tools/primer-blast/; last accessed on 10.04.2017

2 http://eu.idtdna.com/Primerquest/; last accessed on 10.04.2017

## Supplementary Material

**FIGURE S1 | Prunasin** **(A)**, prunasin amide **(B)**, prunasin acid **(C)**, prunasin anitrile **(D)**, amygdalin **(E)**, prunasin pentoside **(F)**, prunasin anitrile pentoside **(G)** and β-D-glucose-1-benzoate **(H)** in flower parts (pistils, petals, and sepals) from five almond cultivars with different flowering times (earliest to latest: Achaak, Desmayo, S3067, Lauranne and Penta) at the flowering time day. Bars indicate standard deviation.
