# Supplementary figures and images for: Cyanogenic Glucosides and Derivatives in Almond and Sweet Cherry Flower Buds from Dormancy to Flowering
Source: Front Plant Sci. 2017 May 19;8:800. doi: 10.3389/fpls.2017.00800 (PMC5437698; doi:10.3389/fpls.2017.00800)

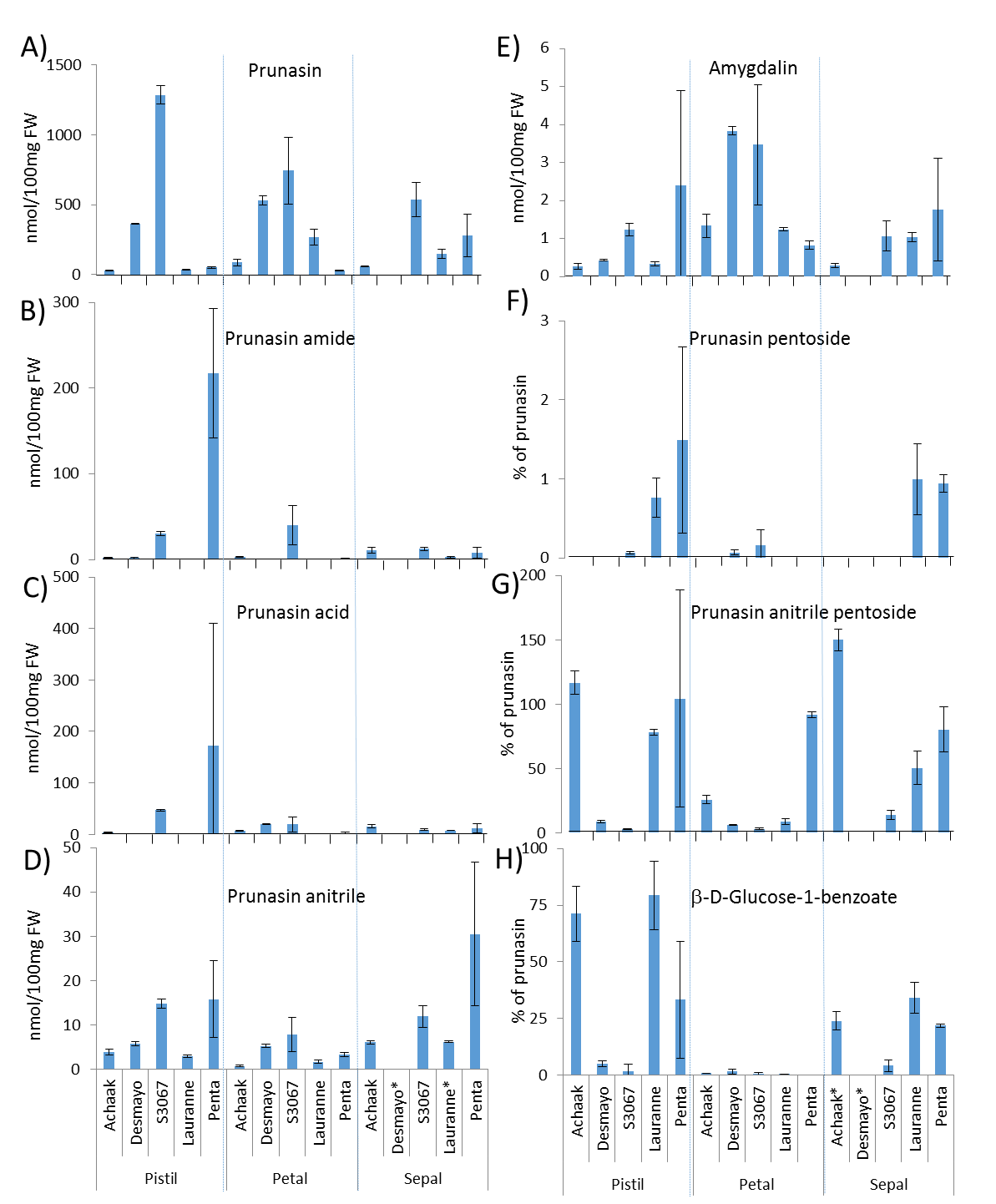

Supplement: Supplementary file 1 [file Image_1.TIF]
