# Supplementary material for: Cyanogenic Glucosides and Derivatives in Almond and Sweet Cherry Flower Buds from Dormancy to Flowering
Source: Front Plant Sci. 2017 May 19;8:800. doi: 10.3389/fpls.2017.00800 (PMC5437698; doi:10.3389/fpls.2017.00800)
Supplement: Supplementary file 2 [file Data_Sheet_1.DOCX]

**List of used coding sequences for qRT-PCR primer design**

*Reference genes*

> 18S rRNA | *Prunus avium*

ATGGTGAGGCCATATGCAGTGAAGGGAAAGAAGCGAAGGAAGAGGGAAGATAAATATGAAAGAGTAGAAGAAGCAGAAGAAGCAGAAGAAGCGTCAGAACCAGAAGAAACAGAGGCTTCACAGCCAGAGGACGTTAACAAGGGAGCTATGGTTGAGAAACAGAATAATGGAAACGAAGGAGAAGAAAAAGTGGGTAACCCAATTGAACTTGAGGGTATTCCAATAACCCCGGTAGAACTTAGCACCAAGAACGGACCTGGGGCCATCTTCATCCTTGAGAGAGCTTCTTTGGAAGTTGCCAAAGTTGGAAAGACTTATCAGCTATTGAATTCTGATGATCACTCAAATTTCTTGCGGAAGAACAACAGAGATCCTGGTCTTTACAGGCCCGATATCGTTCATCAGGCTCTCCTTATGGTTTTGGATAGCCCAATTAATAAAGCTGGGAAGTTGCGAAATGTGTACGTGAGAACTGCAGCAGGTGTTCTTATTGAAATTAAGCCACATGTTCGTATCCCAAGGACATTTAAGCGGTTCTGTGGTGTCATGTTGCAACTGCTACAAAAACTGAGTATTTCTGCTGTTGGTAAGCGTGAGAAACTTATGCGTGTGATAAAGAACCCCGTGACACAGTATTTACCTGTCAACTCTCGCAAAATAGGCTTCTCATACAGTTCAGAAAAATTAGTTAGCATACAGAATTATGTTGCTGCTGCTGAAAACAATAGAGACTTTGTTTTTGTGGTTGGTGCGATGGCTCATGGAAAAGTGGAGACAGACTATACTGATGATTTTATATCAATCTCTGGTTACCCCTTGAGTGCTGCATTTTGTATTTCGATGATTTGCCAAGCATTGGCGGGAAAGTGGGATATTTTGTGA

> Ribosomal protein L13 (RPL13) | *Prunus avium*

ATGGTGAAGCACAACAATGTCATTCCTAGCTCGCACTTCAGAAAGCATTGGCAGAATTATGTGAAAACGTGGTTTAATCAGCCAGCGCGTAAAACACGTCGAAGAAAGGCCCGTCAAGAGAAAGCTGTGAAGATTTTTCCTCGCCCTACATCTGGACCACTGCGACCTGTCGTTCATGGCCAAACGTTAAAATACAATATGAAAGTAAGATCTGGCAGAGGATTTACTCTTGAAGAACTGAAGGCTGCTGGTATCCCGAAGAAGCTTGCTCCAACCATTGGCATAGCTGTTGATCATCGCCGCAAGAATCGATCTCTAGAAGGTCTTCAAGCAAATGTTCAGAGGTTGAAAACATATAAGGCCAAATTAGTTGTCTTCCCAAGACGTGCTGGGAAATTCAAGGCGGGCGATTCAAGTCCTGAGGAGCTTGCCAATGCTACCCAAATTTCAGGCCCTTACATGCCCATTGTAAGGGAGAAGCCAACTGTTGAGCTTGTGAAGGTTACAAATGATATGAAGGCATTTAAGGCTTATGACAAGCTACGCGTTGAGCGTATGAATGAACGTCATGTTGGTGCGAGACTGAAGAAGGCTGCCGAGGCAGAGAAGGAAGAAAAGAAATAG

> Transcription Elongation factor 2 (TEF2) | *Prunus avium*

GGTGGTGGATATGTGTAAGGGTGTTCAGTACCTGAATGAAATTAAGGACTCTGTTGTTGCTGGTTTCCAGTGGGCTTCAAAGGAAGGTGCATTGGCAGAAGAAAACATGAGGGGTATTTGCTTTGAAGTCTGTGATGTGGTTCTTCACGCTGATGCCATCCACAGAGGAGGTGGTCAGGTCATTCCCACTGCTAGGAGGGTCATCTATGCTTCCCAGATCACTGCCAAGCCAAGGCTCCTTGAACCTGTATATCTTGTTGAAATCCAAGCTCCAGAGCAGGCTCTTGGTGGTATCTACAGTGTTCTTAATCAGAAACGTGGGCATGTGTTTGAGGAAATGCAGAGGCCAGGTACTCCACTCTACAATATCAAGGCATACCTCCCCGTCGTTGAATCTTTTGGGTTCTCTGGTCAACTGAGGGCTTCGACTTCAGGTCAGGCCTTCCCACAATGTGTGTTTGATCATTGGGAGATGATGTCGTCTGATCCATTGGAAGCTGGATCCCAGGCTGCACAGCTTGTTACAGATATCCGTAAGAGGAAGGGTTTGAAGGAGCAAATGACCCCACTATCCGAGTTTGAGGACAAACTCTGAGTAATTTCTTTGATTTTATCGAAGAATTGGATGACTGAGTTACTTTGAGACAGTAGTTCCATTGCACTGCCTATTTTTGGTTTTCAGTTTTTTGTTTTCGTTTAATGTTTGTACCAACCCTCCTGTTGTTGTAGTCAGCATCCTGTGCCAGTAGTAACGTTTTGTGCGTGCTCAGAAGATTATGTAATGTTATGTTCTTACTTTGTTGCTTATGGAATCATTTCTGCCATTGCTGGTACATGCTATTTTTGAATTTACTATACACCCCTTTTCATGGTTTGTTTCGTGGATTGGATAGCTTGTTCTTGCATAACTAGACTGTTCTGTGTGTTTGCTACAGCGTCATATCTCAATAGATATAGGAAAGCAGGC

> Peroxidase | *Prunus avium*

ATAAGTAGACGGCATGATTTATTTTCCCCCCCTTTTAGGGAATAGTATCACTAAATATCCACTTATATTATAACTACAAGATGTAGCACTAGTATTCTAGCCATCATCTAGTTCTTTTTATATATATTTCTCCAGCCAACCACACCATTCAATCTCAGTCTCTCATTCAAATTATATAAAGTCCTCTGCTTCTTCTCTTCCCCTGTGTCCTGCTAAATTCAATTTTTGCTCACATGGGTAGGCTCAATCCTATACTTGTTTGGTCGCTTTGCCTCAGTTTGTGCCTCTTTCTCTGTCCCACATCGGCGCAGCTAAAAACAAACTTCTACGCCAACGTCTGCCCCAATGTCGAAAACATTGTAAAAAATGTTGTCACTCAGAAATTTCAACAAACATTTGTCACAGTCCCAGCAACCATCCGTCTCTTTTTTCATGATTGCTTTGTCCAGGGTTGTGATGCTTCGGTTATAGTTGCTTCCACTGGAAACAACAAAGCAGAGAAGGATCATCCAGATAATCTGTCATTAGCTGGAGATGGTTTTGATACAGTGATCAAAGCCAAAGCAGCAGTTGATGCAGTTCCTCAGTGCAAAAACAAAGTCTCATGTGCTGATATTCTTGCCTTGGCCACCAGAGATGTCATTGTTCTGTCTGGTGGACCTTCGTATGCTGTTGAGTTGGGGAGATTGGACGGATTAATTTCAAGTTCTAAAAATGTTGATGGGAACCTCCCCCAGCCAACCTTCAATTTGAACCAGCTCAATTCCATGTTTGCTTCCCATGGGTTGTCCCAGGCTGACATGGTTGCTCTTTCAGCGGCGCACACCGTTGGATTCTCTCATTGCAACAAGTTTTCCAATCGGATTTACAGCTTCAGTCCCGGGAACCCAGTGGACCCCAGTCTGAACAAAGCATATGCAACTCAACTCCAACAGATGTGTCCAAAAAATGTGGACCCCGACATAGCCATCAACATGGACCCAAACACGCCCAGAACCTTTGACAACGTCTATTTCAAGAATCTTGAACTTGGGCAAGGCCTTTTTACCTCAGACCAGGTCCTTTTCACGGATGCAAGGTCTCAGCCCACGGTAAAAACCTGGGCCAAAGACAACGCTGCTTTTCAACAGGCTTTTACCACCGCGATGACCAAGCTGGGCCGGGTCGGAGTCAAGACAGGCAAAAACGGAAATATTCGTAGCGATTGTAGTGTTTTTAACTGAGAAGGAAATTTTTGGGTTAAAAAAATATATATATATATATTTGAAGGTAAAACTGAATGTTGACTAAATTCATTTGACATTTTACTCCTTTCTTTTTCCTTCTTTCTTTTATCATTTGAAGGTAAAACTATCACTGGTTTGTTATTAGAGTCGTGCAATTATTAGAAATCAATTTGTTCACATGAAGGCCACAATAATTTCCAGATTCTCGTTATAAATTAATTTGCAGAAAAAAAGACTTTTGACTGTGTCTTGCATTATTTTAGAAGTCTTTCTTTTGTGGTCATGTAGACCCCAACGCTGAGTCATTTGAAGGAGATTTTATTTTTCTTTCTTGTTTCTTTTGAGAGAGGAGGTTTGATTTTTCAAGATGCTTATAGCTTGTCATTATCCTCCAGACTTTCTACTGTTCCTTGATGGTTTCCTGTATGCATCATTGTTTGCTAATCCTCAACTTAATCTGGTGATAAGACCCTCTCCCACCAATCCTTGTTTGCTAATCTTCATCTTAATTTGGTG
